# Register-based study of the incidence, comorbidities and demographics of obsessive-compulsive disorder in specialist healthcare

**DOI:** 10.1186/s12888-017-1224-3

**Published:** 2017-02-10

**Authors:** Hanna Rintala, Roshan Chudal, Sami Leppämäki, Susanna Leivonen, Susanna Hinkka-Yli-Salomäki, Andre Sourander

**Affiliations:** 1 Department of Child Psychiatry, University of Turku, Turku, Finland; 2 Department of Psychiatry, Helsinki University Central Hospital, Helsinki, Finland; 3 Department of Child Neurology, Helsinki University Central Hospital, Helsinki, Finland; 4 Department of Child Psychiatry, University of Turku and Turku University Central Hospital, Turku, Finland; 5 Research Centre for Child Psychiatry, Institute of Clinical Medicine, Faculty of Medicine, University of Turku, Lemminkäisenkatu 3 / Teutori (3rd floor), 20014 Turku, Finland

**Keywords:** Obsessive-compulsive disorder, Time trends of incidence, Comorbidity, Mental health services, Register-based study

## Abstract

**Background:**

Incidence of obsessive-compulsive disorder (OCD) has been suspected to increase but nationwide epidemiological studies are limited. This study aims to examine sex-specific incidence time trends and characterize psychiatric and neurodevelopmental comorbidities and sociodemographic risk factors of OCD in specialist healthcare in Finland.

**Methods:**

A nationwide register-based study using data from four Finnish registers identified 3372 OCD cases and 13,372 matched controls (1:4). Cumulative incidence in subjects born between 1987 and 2001 was estimated at ages of 10, 15, 20 and 23 years. Conditional logistic regression was used to examine the sociodemographic factors.

**Results:**

The cumulative incidence of OCD was 0.4% by age 23. Incidence by age 15 among three cohorts increased from 12.4 to 23.7 /10000 live born males and 8.5 to 28.0 /10000 live born females. 73% of the sample had a comorbid condition. Males were significantly more comorbid with psychotic and developmental disorders; females were more comorbid with depressive and anxiety disorders (*p* <0.001). Higher maternal SES was associated with an increased risk of OCD (OR 1.4; 95% CI 1.1–1.6).

**Conclusions:**

These findings suggest that incidence of treated OCD in specialist healthcare has increased. The reason may be increased awareness and rate of referrals but a true increase cannot be ruled out. Further research on risk factors of OCD is warranted.

## Background

Obsessive-compulsive disorder (OCD) is an often disabling and chronic psychiatric condition that affects people throughout their lives [1–3]. It is characterised by obsessive, intrusive and anxiety-provoking thoughts and compulsive, repetitive behaviours that aim to reduce the anxiety related to obsessions. Transient obsessions and compulsions are a common human phenomenon, but to fulfil the diagnostic criteria of OCD, the symptoms have to be time-consuming or cause significant distress or functional impairment [1]. OCD typically starts at a young age the age of onset peaking at 11 and 23 years, and follows a chronic course with varying symptom severity [2, 4–6]. OCD is often under-diagnosed and considerable diagnostic delays have been reported [2, 7].

OCD is relatively common with an estimated lifetime prevalence of 1–2%, with similar global patterns [2, 8–14]. Register-based multigenerational and twin studies in Sweden and Denmark showed that OCD had a strong familial pattern [15–17]. Most previous epidemiological studies have been cross-sectional prevalence studies with very different methodologies, ranging from population-based screening studies to clinical studies. Therefore, it is difficult to compare their findings and draw conclusions about changes in incidence of new cases over time. A Danish register-based study reported no increase in OCD incidence in 2007 [18]. Later a similar register study showed an increase of 100–667% in the incidence of OCD in different Nordic countries during the 10 years of follow-up, but did not report gender-specific findings [19].

Comorbidity with other psychiatric and neurodevelopmental disorders is common in OCD and rates between 67 and 92% have been reported in clinical and population screening studies [20–24]. The most common comorbidities include anxiety and mood disorders, psychotic disorders, attention deficit hyperactivity disorder (ADHD) and tic disorders [5, 20–24]. Comorbidity with tic disorders has particularly been associated with male predominance [22–24]. Up until now there are no register-based studies reporting overall gender-specific comorbidities in OCD.

A recent systematic review of environmental risk factors of OCD listed only few studies on sociodemographic factors with limited quality [25]. Previous studies of the associations between parental socioeconomic status (SES) and OCD have shown inconsistent findings and none of these have been register-based studies [8, 11, 14, 26–29]. Specifically low parental education and SES have previously been linked to mental disorders [30, 31], but OCD was not addressed in these studies. Despite sociodemographic changes in recent decades, such as higher standard of living and urbanization development, these factors and OCD have rarely been addressed. In a Danish register study, living in an urban area was not associated with OCD [17].

Up until now, sex-specific incidence time trends in treated OCD subjects have not been reported in nationwide register studies. The main objective of this study was to examine the OCD incidence in mental healthcare services during a total of 25 years’ follow-up and assess the possible sex differences. Our second aim was to examine the incidence of psychiatric and neurodevelopmental comorbidity in the subjects during the follow-up time. Third aim was to describe the association between maternal SES, degree of urbanity and OCD in offspring.

## Methods

This study was based on a nested case-control design and the source population included all live births in Finland between 1 January 1987 and 31 December 2009 (*N* = 1 390 172). Data were obtained from four Finnish nationwide registers: The Finnish Hospital Discharge Register (FHDR), The Finnish Central Population Register (FCPR), The Finnish Medical Birth Register (FMBR) and Statistics Finland. Detailed information of these registers has previously been described and is only briefly described here [32, 33].

The present study used the FHDR to identify individuals with OCD referred to specialist mental healthcare. All inpatient admission and discharge dates and primary and subsidiary diagnoses at discharge have been recorded in the FHDR since 1969 and since 1998 it has also recorded all outpatient episodes in public specialist healthcare. Until the end of 1995, the diagnoses were coded according to the International Classification of Diseases, Ninth Revision (ICD-9) and from 1996 the register used the Tenth Revision (ICD-10). Attending physicians in public specialist healthcare provide the information in the FHDR. OCD diagnosis in Finland is based on ICD-10 symptom criteria. The overall validity of diagnoses in the FHDR has been systematically reviewed in 2012 and the positive predictive value was found to be 75–97% [34].

The FCPR was used to collect information on the demographic characteristics of the individuals, and it provides information on citizenship, family members, place of residence and dates of birth and death. The FMBR contains detailed data on all pregnancies, births and neonatal periods until 7 days of age and since 1991, it has also contained data on maternal socioeconomic status. The study was authorised by the Ministry of Social Affairs and Health (STM/1528/2007) and the National Institute of Health and Welfare (all registers mentioned above). The access to the databases is limited in the study conditions. The Ethics Committee of the Hospital District of Southwest Finland provided ethical approval for the study.

### Cases and controls

Cases were defined as individuals diagnosed with OCD and registered in the FHDR by the end of 2012. The ICD-9 code 3003A was used until the end of 1995 and the ICD-10 codes F42.0, F42.1, F42.2, F42.8 and F42.9 were used from the beginning of 1996. We excluded one case with severe or profound mental retardation, two sets of multiple births and three children diagnosed with OCD only before the age of two. The date of the first OCD diagnosis was used for identification. We identified four controls from the Population Register for every OCD case and these were individuals born during the study period without any diagnosis of OCD or any anxiety disorder (ICD-10 codes F40, F41) in the Discharge Register. They were matched to the case by sex, date of birth (±30 days) and were living in Finland at the time that the matched case received their first OCD diagnosis. We excluded eight controls with severe or profound mental retardation and 11 sets of multiple births. There were a total of 3372 cases and 13,372 controls in the study.

### Incidence time trends and comorbidity rates of treated OCD

The cases born between 1987 and 2001 were grouped into cohorts of three consecutive birth years: 1987–1989, 1990–1992, 1993–1995, 1996–1998 and 1999–2001. Cumulative incidence was defined as the number of new OCD subjects per 10000 live births by the ages of 10, 15, 20 and 23 years in each cohort. The cumulative incidence includes all subjects in the cohort diagnosed by the observation age. Comorbid psychiatric and neurodevelopmental diagnoses of the subjects recorded during the follow-up period were identified in the FHDR according to the ICD-9 and ICD-10 codes. All comorbid diagnoses were assessed by classifying them into similar groups as in previous register studies [35, 36]: chronic tic disorder or Tourette syndrome (ICD-9 3072, which includes all tic disorders and ICD-10 F95.1, F95.2 including chronic tic and Tourette), ADHD or ADD/Attention deficit (hyperactivity) disorder (ICD-9 314X and ICD-10 F90, F98.8), conduct disorders (ICD-9 3120 and ICD-10 F91), autism spectrum disorders (ICD-9 299X and ICD-10 F84), developmental disorders (ICD-9 3153, 3154 and ICD-10 F80, F81, F82, F83), mild or moderate mental retardation (ICD-9 3170, 3180 and ICD-10 F70, F71), eating disorders (ICD-9 3071, 3075 and ICD-10 F50), anxiety disorders (ICD-9 300X and ICD-10 F40, F41), depressive disorders (ICD-9 296X, 3004 and ICD-10 F32, F33, F34), bipolar disorder (ICD-9 296X and ICD-10 F30, F31) and psychotic disorders (ICD-9 295, 2971, 298 and ICD-10 F20, F22, F23, F25, F28, F29).

### Parental demographic characteristics

Data on maternal socio-economic status (SES), based on the occupation of the mother, were obtained from the FMBR from 1991 onwards. Paternal SES is not collected in the register, and maternal SES variable has been categorised into four classes. The classes are upper white collar (e.g. professional and administrative), lower white collar (e.g. office labourer), blue collar (manual labourer) and others (e.g. students, unemployed and entrepreneurs). These have been previously described in more detail [37, 38]. Data on residential area at birth was collected from the FCPR, which provided information on the region of birth and urbanity. The region of birth of the subjects with OCD was examined by dividing the country into southern, western, northern and eastern Finland. Areas were defined as urban, semi-urban or rural based on the classifications provided by Statistics Finland and the details have previously been described [39, 40]. The covariates selected for the study included: cohort effect, maternal psychopathology and maternal age. The cohort effect was examined by dividing the sample into three birth periods (1991–1994, 1995–1998, 1999–2008), maternal age was divided into age ranges (<19 years, 20–24, 25–29, 30–34, 35–39 or >40 years) and maternal psychiatric history was examined as a dichotomous variable (yes or no). Since the FMBR was established in 1991, information on parental demographic characteristics was available only for 2241 cases (66%) and 8794 (66%) controls.

### Statistical analyses

Cumulative incidence per 10,000 live births and the corresponding 95% confidence intervals of diagnosed OCD were estimated with a Poisson-regression model, assuming a Poisson error distribution. Comorbidity frequencies and percentages among the sample were calculated separately for males and females. The gender differences were tested with Pearson’s chi-square test.

Bivariate analysis was conducted to test for the association between potential covariates and risk factors in the controls and in relation to OCD. The significant covariates were selected for the adjusted analysis. Associations between maternal SES, urbanity and region of birth and OCD in the offspring were analysed with conditional logistic regression analysis. The results of these analyses are presented as odds ratios (OR) with 95% confidence intervals (95% CI). For testing, we used two-sided *p*-values at a 0.05 significance level. The statistical analyses were performed with SAS statistical software version 9.4 (SAS Institute Inc., Cary, North Carolina, USA).

## Results

There were a total of 3372 OCD subjects treated in specialist health care in the study. Of the total, 1579 (47%) were males and 1793 (53%) were females. The mean age at OCD diagnosis was 15.2 years, with a standard deviation (SD) of 4.1 and a range of 3–25 years. The mean age among males and females was 14.8 years (SD 4.3, range 4–25 years) and 15.6 years (SD 3.9, range 3–25 years), respectively. Of the covariates, maternal psychopathology and maternal age were associated with both the risk factors and OCD (*p* < 0.1).

**Fig. 1:**
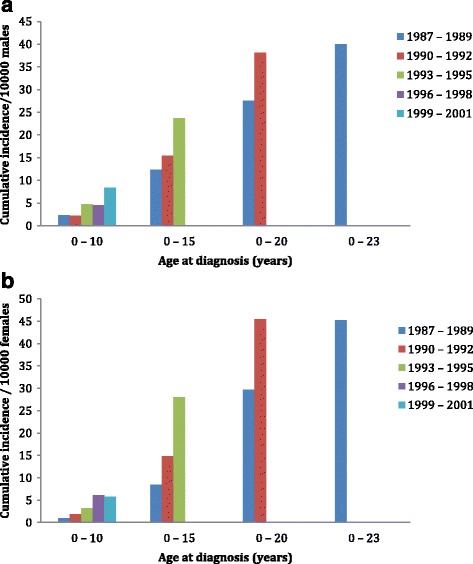
**a** Cumulative incidence per 10,000 live born males according to birth year. **b** Cumulative incidence per 10,000 live born females according to birth year

Figure 1a and b shows the number of new cases per 10,000 live births in the cohorts for males and females. The cumulative incidence of all individuals born in 1987–89 and treated for OCD by the age of 23 years was 0.4%. There was an increasing trend in incidence among both males and females. Incidence by the age 15 increased from 12.4 to 23.7 /10,000 live born males and from 8.5 to 28.0 /10,000 live born females between the cohorts 1987–89 and 1993–95. The gender difference in incidence by age 15 was significant in the 1987–89 cohort (*p* < 0.01). There were few individuals diagnosed at younger than 10 years of age. During the follow-up, incidence by 10 years among males increased from 2.3 (born 1987–89) to 8.4 (born 1999–2001)/10,000 and among females from 1.0 (born 1987–89) to 5.8 (born 1999–2001)/10,000. Gender differences in incidence by age 10 were significant in the 1987–89 and 1999–2001 cohorts (*p* < 0.01).

Table 1 shows comorbidities among OCD cases, separately for males and females. Overall, 70.8% of males and 74.8% of females were diagnosed with at least one additional psychiatric or neurodevelopmental disorder and the most common comorbidities were anxiety disorders (males 32.8%, females 46.5%), depressive disorders (males 29.8%, females 46.3%) and psychotic disorders (males 15.3%, females 11.3%). There were significant gender differences in several comorbidities. Anxiety, depressive and eating disorders were more common among females (*p* <0.001), whereas psychotic, developmental and autism spectrum disorders and ADHD/ADD were more common among males (*p* <0.001).

**Table 1:** Gender differences in comorbidity among OCD cases in specialist mental healthcare

Comorbidities	Total		Male		Female		OR (95% CI)	*p* value
	*n*	%	*n*	%	*n*	%		
Any comorbidity	2459	72.9	1118	70.8	1341	74.8	1.22 (1.05–1.42)	0.01
Anxiety disorder	1352	40.1	834	32.8	518	46.5	1.78 (1.55–2.05)	<0.001
Depressive disorder	1301	38.6	471	29.8	830	46.3	2.03 (1.76–2.34)	<0.001
Psychotic disorder	445	13.2	242	15.3	203	11.3	0.71 (0.58–0.86)	<0.001
Developmental disorder	404	12.0	278	17.6	126	7.0	0.35 (0.28–0.44)	<0.001
Eating disorder	324	9.6	49	3.1	275	15.3	5.66 (4.14–7.73)	<0.001
ADHD + ADD	267	7.9	191	12.1	76	4.2	0.32 (0.24–0.42)	<0.001
ASD	271	8.0	211	13.4	60	3.4	0.22 (0.17–0.30)	<0.001
Tic disorder	151	4.5	118	7.5	33	1.8	0.23 (0.16–0.34)	<0.001
Bipolar disorder	125	3.7	42	2.7	83	4.6	1.78 (1.22–2.59)	0.003
Conduct disorder	117	3.5	66	4.2	51	2.8	0.67 (0.46–0.97)	0.04
Mental retardation	40	1.2	22	1.4	18	1.0	0.72 (0.38–1.34)	0.34

*ADHD* attention deficit hyperactivity disorder, *ADD* attention deficit disorder, *ASD* autism spectrum disorder, *OR* odds ratios

In the unadjusted analysis, high maternal SES, living in an urban area and being born in southern or eastern Finland were all associated with an increased risk of OCD. These are shown in Table 2. Children whose mothers were from the upper white collar SES class were significantly associated with higher rates of referrals because of OCD (OR 1.36, 95% CI 1.15–1.59, *p* <0.001), compared to children born to blue collar workers. The finding remained significant when it was adjusted for maternal psychiatric history and maternal age (OR 1.26, 95% CI 1.07–1.49, *p* = 0.007). Subjects born in urban areas had significantly elevated rates of OCD compared to those born in rural or semi-urban surroundings (OR 1.42, 95% CI 1.26–1.61, *p* <0.001). This finding also remained significant in the adjusted analysis (OR 1.29, 95% CI 1.13–1.47, *p* <0.001).

**Table 2:** Demographic factors and risk of OCD

						Unadjusted			Adjusted^a^		
		Cases (n)	%	Controls (n)	%	OR	95% CI	*P* value	OR	95% CI	*P* value
Maternal socioeconomic status	Blue collar (ref)	363	16.4	1639	18.6	Ref					
	Upper white collar	389	17.5	1296	14.7	**1.36**	**1.15–1.59**	**<0.001**	**1.26**	**1.07–1.49**	**0.007**
	Lower white collar	1018	45.9	3963	45.1	1.16	1.01–1.32	0.03	1.14	1.00–1.31	0.06
	Others	336	15.2	1444	16.4	1.05	0.89–1.24	0.54	1.04	0.88–1.24	0.62
	Missing	112	5.1	452	5.1	1.12	0.88–1.42	0.36	1.07	0.83–1.38	0.63
Residential area	Rural (ref)	378	17.0	1926	21.9	Ref					
	Urban	1483	66.9	5314	60.4	**1.42**	**1.26**–**1.61**	**<0.001**	**1.29**	**1.13**–**1.47**	**<0.001**
	Semi-urban	340	15.3	1513	17.2	1.14	0.97–1.34	0.12	1.10	0.93–1.30	0.27
	Missing	17	0.8	41	0.5	2.08	1.17–3.70	0.01	1.95	1.04–3.64	0.04
Region of birth	Northern Finland (ref)	222	10.0	1184	13.5	Ref					
	Southern Finland	1119	50.5	3560	40.5	**1.68**	**1.43**–**1.96**	**<0.001**	**1.49**	**1.27**–**1.76**	**<0.001**
	Western Finland	624	28.1	3097	35.2	1.08	0.91–1.27	0.39	1.04	0.88–1.24	0.63
	Eastern Finland	253	11.4	953	10.8	**1.42**	**1.16**–**1.73**	**<0.001**	**1.36**	**1.11**–**1.67**	**0.003**

*OR* odds ratio, *Ref* reference category, significant findings in boldface

^a^Adjusted with maternal age and maternal psychiatric history

Missing 1154 cases

## Discussion

The first finding was an increasing trend in the number of subjects treated in specialist healthcare because of OCD during the study period. The change was similar for both males and females. An earlier study carried out in Denmark in 2007 suggested that the trend in the incidence of OCD had not increased [18], whereas an increasing trend in OCD was observed in a multinational comparison study of incidence time trends in childhood neuropsychiatric disorders [19]. The observed increase was thought to be due to improvements in service availability and an increase in help-seeking behaviour. Of note, the use of mental health services by Finnish children increased three-fold from 1989 to 2005 and a similar trend was observed in the Netherlands [41, 42]. This has certainly been reflected in the increasing incidence of OCD in the referred population.

Another possibility for increased treated incidence is that there was a real increase in the incidence of OCD during the follow-up period. Explanations for the real increase of OCD incidence could be hypothesised to fall into the same broad spectrum of risk factors as other childhood onset psychiatric and neuropsychiatric disorders. These have been studied widely with regard to autism spectrum disorders and ADHD, but not to OCD. Background factors for the possible true increase in the incidence of OCD may include prenatal exposure to environmental toxins [43], medications during pregnancy [44], adverse prenatal or perinatal events [45, 46], advanced parental age [17, 47] and parenting and lifestyle related factors [25, 48]. Infections and autoimmune mechanisms have also been suggested to underlie the increasing incidence of OCD, particularly paediatric autoimmune neuropsychiatric disorders associated with the streptococcal infections subtype [49]. A recent systematic review did not reveal any strong candidate for environmental risk factors specific for OCD, and the general quality of the studies involved was considered limited [25].

Secondly, we found that people treated in specialist healthcare for OCD had a very high incidence of psychiatric and neurodevelopmental comorbidities and the finding was in line with previous findings of comorbidities in OCD [2, 20, 21]. This finding reflected the accumulation of psychiatric and neurodevelopmental symptoms among individuals [15, 16, 50]. High levels of comorbidities may also be related to the internalising nature of OCD and to the problems of awareness and recognition of the disorder, as many individuals may be diagnosed with other psychiatric or neurodevelopmental disorders before OCD symptoms are detected [7, 51]. High comorbidity rates also reflect the problem with symptom-based diagnostic systems and the complex nature of psychiatric and neurodevelopmental symptoms. There were differences between males and females in our study with regard to OCD comorbidities, as males with OCD were more likely to have a comorbid diagnosis of externalising disorders such as ADHD, whereas females were more often comorbid with anxiety and depressive disorders, which are considered internalising. This is in line with previous reports [22, 23].

Thirdly, high maternal socioeconomic status and being born in an urban area were associated with more frequent referrals to mental health services because of OCD, but living in northern Finland was associated with a lower rate of referrals. This possibly reflects regional and social class differences in service accessibility and differences in awareness between individuals to seek counseling. Generally low social class or low education have been linked to adverse mental health and although Finland and other Nordic countries are known for their relatively low inequality between social classes, there has been public worry about growing inequalities [30, 31, 52, 53]. Public mental health services are free of charge and available for everyone, which means that the high SES group was not overrepresented in the referred population because of issues with affordability [30, 31]. It has been speculated that such inequalities exist in many countries, including the US, where healthcare is funded by private insurances [54, 55]. It has been suggested that the uneven distribution of diagnostic and treatment services is a universal phenomenon [56, 57]. OCD tends to be more internalising than many other psychiatric and neurodevelopmental disorders that start at a young age and there are often obstacles to diagnosis and treatment [57, 58]. Parents in the high SES group seemed to be more likely to have their children diagnosed, but the pathway was not clear. Differences between regional and urban and rural areas may have reflected the uneven distribution of healthcare services targeted at OCD.

The limitations of the study need to be considered when evaluating these findings. Due to our study design, some cases may have been missed because of inadequate follow-up. The subjects in the latest birth cohorts were not likely to have reached the average age at diagnosis when the follow-up period ended. Some subjects in the earliest cohorts may have been missed, since the outpatient visits were only recorded in the FHDR from 1998 onwards. The exclusion criteria for the control subjects contained any recorded anxiety disorder because the OCD data were part of a larger anxiety disorder data collection. Therefore the risk factors may not be specific to OCD but may also apply to various other disorders, which we were not able to investigate at this stage.

## Conclusions

This study shows an increasing incidence of treated OCD in specialist healthcare. This reflects increased rate of referrals although an actual increase in the incidence of OCD cannot be ruled out. There is still need for further awareness of OCD diagnosis and research on risk factors. This Finnish OCD sample provides an opportunity to examine a range of prenatal and perinatal risk factors of OCD in the future.

## Abbreviations

ADD: Attention-deficit disorder
ADHD: Attention-deficit/hyperactivity disorder
ASD: Autism spectrum disorders
CI: Confidence interval
FCPR: Finnish Central Population Register
FHDR: Finnish Hospital Discharge Register
FMBR: Finnish Medical Birth Register
ICD: International Classification of Diseases
OCD: Obsessive-compulsive disorder
OR: Odds ratio
SES: Socioeconomic status
